# Correction: Beyond survival: The impact of birth complications on postpartum wellbeing for mothers, babies, and households in Kenya

**DOI:** 10.1371/journal.pgph.0006536

**Published:** 2026-05-26

**Authors:** Laura Subramanian, Dorothy Oluoch, Michuki Maina, Edna Mutua, Lauren Bobanski, Madison Canfora, Joyline Jepkosgei, Juliet Jepkosgei, Pauline Karingu, Consolata Chesang, Justinah Maluni, Sarah Dannin, Ki-Do Eum, Mike English, Ami Karlage, Felistas Makokha, David Kimutai, Kalama Fondo, Dickens Lubanga, Katherine E. A. Semrau, Danielle E. Tuller, Sassy Molyneux, Megan Marx Delaney

The current address of “Institute of Tropical Medicine, Antwerp, Belgium” for author Justinah Maluni is incorrect. The affiliation “Health Services Unit, KEMRI-Wellcome Trust Research Programme, Nairobi, Kenya” is the correct and sole affiliation for this author at the time of study.
