# Supplementary material for: Beyond survival: The impact of birth complications on postpartum wellbeing for mothers, babies, and households in Kenya
Source: PLOS Glob Public Health. 2025 Dec 5;5(12):e0004845. doi: 10.1371/journal.pgph.0004845 (PMC12680264; doi:10.1371/journal.pgph.0004845)
Supplement: S1 Checklist — (DOCX) [file pgph.0004845.s003.docx]

Inclusivity in global research

PLOS’ policy on inclusivity in global research aims to improve transparency in the reporting of research performed outside of researchers’ own country or community and ensures that PLOS publications reporting global research adhere to high standards for research ethics and authorship. Authors of relevant research articles may be asked to complete the questionnaire below, which outlines ethical, cultural, and scientific considerations specific to inclusivity in global research. This questionnaire may be requested when researchers have travelled to a different country to conduct research, if research uses samples collected in another country, research with Indigenous populations or their lands, or if research is on cultural artefacts. Researchers travelling to another country solely to use laboratory equipment will not normally be required to complete the questionnaire. However, the questionnaire can be requested at the journal’s discretion for any submission – if you have been requested to complete this questionnaire by the PLOS journal you submitted to, please do so.

Please complete the questionnaire below and include this as a Supporting Information file with your manuscript. Note that if your paper is accepted for publication, this checklist will be published with your article in the supporting information files. Please ensure that you reference the checklist in the main body of your manuscript. We suggest adding a subsection ‘Inclusivity in global research’ to your Methods section and adding the following sentence: “Additional information regarding the ethical, cultural, and scientific considerations specific to inclusivity in global research is included in the Supporting Information (SX Checklist)”

The questions have been designed to be applicable to a wide range of study types, and there are subsections for both human subjects research and non-human subjects research. If any of the questions are not relevant to your research please mark them as “N/A” as appropriate.

**Ethical considerations, permits and authorship**

*This section is applicable to all research types.*

Provide details as to who granted permissions and/or consent for the study to take place in the Methods section of your manuscript. This should include the names of **all** ethics boards, governmental organizations, community leaders or other bodies that provided approval for the study. If individuals provided approval refer to these people by their role or title but do not list their name(s).

Reported on page number: 12. All of the ethics boards and governmental organizations that granted permissions for the study are listed in lines 221-225 of the manuscript.

If there were any deviations from the study protocol after approval was obtained please provide details of these changes in the Methods section of your manuscript.

Reported on page number: 12. Prior to starting data collection, we reduced the number of study sites from four to three. This was an operational change due to feasibility and resource constraints, and we obtained amendments accordingly from all of the relevant ethics committees and review boards.

Did this study involve local collaborators that are residents of the country where the research was conducted or members of the community studied? If you do not have any authors from said communities, please provide an explanation for this below.

This study was co-led by researchers at KEMRI-Wellcome Trust Research Programme (a local research organization based in Kenya) and Ariadne Labs (based in Boston, MA). Several KEMRI-Wellcome Trust researchers are authors on the manuscript, including the co-first author Dr. Dorothy Oluoch. Focal persons at the study hospitals who are employed by the respective County Governments for each study site were also involved in the study and are coauthors on the manuscript.

Everyone listed as an author should meet PLOS’ criteria for authorship and all individuals who meet these criteria should be included in the author byline, rather than the acknowledgements. For further information please see the journal’s Authorship Policy.

**Human subjects research (e.g. health research, medical research, cross-cultural psychology)**

Did you obtain written informed consent from a representative of the local community or region before the research took place? How did you establish who speaks for the community? Details of written informed consent obtained from study participants should be reported separately in the Methods section of your manuscript.

As per the requirements, we obtained approvals from the respective County Departments of Health, which are responsible for granting permission to conduct studies within their jurisdictions. Once these approvals were secured, we proceeded to engage with the management teams of the selected hospitals. For Nairobi and Bungoma, we conducted introductory engagement meetings with hospital staff and mothers in the recruitment wards. In Kilifi, where KEMRI-Wellcome Trust Research Programme has an established presence through long-standing research activities and a demographic health surveillance system, we followed the existing community engagement strategy. This included consultations with the existing programme's local community representative group, the hospital management team, and frontline healthcare staff.

Written informed consent procedures for study participants are reported in the Methods section of the manuscript (page 12).

How did members of the local community provide input on the aims of the research investigation, its methodology, and its anticipated outcome(s)?

We engaged with caregivers/mothers and the Community representative group, providing them with details of the research objectives, proposed methods, and expected outcomes. Their input helped refine aspects of the study design, such as participant recruitment strategies. Study hospitals’ management teams and frontline staff were invited to feedback meetings. In these forums, we presented our preliminary findings. They provided feedback on the presented findings, and the study team was able to review the results. We received feedback on areas where more clarity and better framing were needed, as well as further explanations for the observations. We also received input on areas for further research. Throughout the study, regular feedback loops were maintained to ensure ongoing progress.

When engaging with the local community, how did you ensure that the informed consent documents and other materials could be understood by local stakeholders?

The consent forms were reviewed by the KWTRP’s Consent and Communications Committee to ensure that the language in the consent form was simple, easy to understand, and devoid of technical terms. The study's consent forms were also translated into Kiswahili.

Will the findings of the research be made available in an understandable format to stakeholders in the community where the study was conducted (e.g. via a presentation, summary report, copies of publications, etc.)? Please provide details of how this will be achieved.

The study findings have been made available to stakeholders at the study sites in Bungoma, Kilifi and Mbagathi. The KEMRI team went in person to each study site to share the results via presentations, and facilitated discussions with study site staff about the findings. KEMRI will also hold similar meetings with participating mothers and their families to share feedback in a discussion-based format. Additionally, information leaflets will be provided with the study findings.

**Non-human subjects research using specimens/ animals collected as part of the study, or those housed in archival collections. Examples include archaeology, paleontology, botany and zoology.**

Did the permission you obtained from a local authority to perform the study include an agreement on access to outputs and benefit sharing? This may include procedures to enable fair distribution of the benefits and resources arising from the research performed. Please include any details of Prior Informed Consent and Benefit Sharing Agreements obtained. These may be required by field-specific regulations, for example the Convention on Biological Diversity (CBD) and the associated Nagoya Protocol.

N/A

If the material used in your study was imported, please A) provide the year it was imported and B) indicate whether permits were obtained to import/export the materials used, C) provide details of any permits obtained. If this information is not available, please indicate this.

N/A

If you used archival specimens, please state how the material used in your study was acquired by the institute it is held in and provide details of any permits obtained for the original excavations/ sample collection. If this information is not available, please indicate this.

N/A

How was the potential cultural significance of the materials collected in your study to local communities considered in your research design? Were Indigenous peoples and/or local researchers and institutions involved with archaeological excavations / collection of specimens? If so, please provide a description of their involvement.

N/A

If your manuscript includes photographs of human remains please indicate whether authors obtained permission from descendants or affiliated cultural communities to do so.

N/A
